# Dietary Intake and Nitrogen Balance in British Army Infantry Recruits Undergoing Basic Training

**DOI:** 10.3390/nu12072125

**Published:** 2020-07-17

**Authors:** Shaun Chapman, Alex J. Rawcliffe, Rachel Izard, Kimberley Jacka, Hayley Tyson, Lee Smith, Justin Roberts

**Affiliations:** 1 HQ Army Recruiting and Initial Training Command, UK Ministry of Defence, Upavon, Wiltshire SN9 6BE, UK; alex.rawcliffe103@mod.gov.uk (A.J.R.); kimberley.jacka100@mod.gov.uk (K.J.); h.c.tyson@2020.lmju.ac.uk (H.T.); 2 Cambridge Centre for Sport and Exercise Sciences, School of Psychology and Sport Science, Anglia Ruskin University, East Road, Cambridge CB1 1PT, UK; lee.smith@anglia.ac.uk (L.S.); justin.roberts@anglia.ac.uk (J.R.); 3 Defence Science and Technology, Porton Down, UK Ministry of Defence, Salisbury, Wiltshire SP4 0JQ, UK; Rachel.izard715@mod.gov.uk

**Keywords:** dietary intake, protein, training, nitrogen balance, military

## Abstract

We assessed dietary intake and nitrogen balance during 14 weeks of Basic Training (BT) in British Army Infantry recruits. Nineteen men (mean ± SD: age 19.9 ± 2.6 years, height: 175.7 ± 6.5 cm, body mass 80.3 ± 10.1 kg) at the Infantry Training Centre, Catterick (ITC(C)) volunteered. Nutrient intakes and 24-h urinary nitrogen balance were assessed in weeks 2, 6 and 11 of BT. Nutrient intake was assessed using researcher-led weighed food records and food diaries, and Nutritics professional dietary software. Data were compared between weeks using a repeated-measures analysis of variance (ANOVA) with statistical significance set at *p* ≤ 0.05. There was a significant difference in protein intake (g) between weeks 2 and 11 of BT (115 ± 18 vs. 91 ± 20 g, *p* = 0.02, ES = 1.26). There was no significant difference in mean absolute daily energy (*p* = 0.44), fat (*p* = 0.79) or carbohydrate (CHO) intake (*p* = 0.06) between weeks. Nitrogen balance was maintained in weeks 2, 6 and 11, but declined throughout BT (2: 4.6 ± 4.1 g, 6: 1.6 ± 4.5 g, 11: −0.2 ± 5.5 g, *p* = 0.07). A protein intake of 1.5 g·kg^−1^·d^−1^ may be sufficient in the early stages of BT, but higher intakes may be individually needed later on in BT.

## 1. Introduction

British Army basic training (BT) courses include the Combined Infantry Course (CIC) at the Infantry Training Centre, Catterick (ITC(C)) and the Common Military Syllabus (CMS) at the Army Training Centre, Pirbright (ATC(P)). Both courses are characterised by high daily physical demands [1,2] which contribute to high rates of musculoskeletal injuries in recruits [3,4,5]. High daily physical demands combined with other risk factors such as high mechanical loading, sleep restriction, psychological stress, smoking and poor vitamin D status likely exacerbate injury risk in recruits [6]. Men undergoing the CMS have a greater incidence rate of injury compared to their CIC counterparts (417 vs. 391 injuries per 1000 recruits) [6]. The high incidence rates of injury in both courses has a detrimental effect on recruit wastage and organisational effectiveness [5]. Basic training aims to improve aerobic fitness, muscle strength and endurance [4,7], however, maximum dynamic lift strength has been shown to decrease (71 ± 12 vs. 68 ± 11 kg; *p* < 0.01) across 14 weeks of BT in men [8].

The underpinning mechanism for the lack of strength adaptation is likely to be multifactorial. A contributing factor, however, may be an inadequate energy or macronutrient intake to meet training demands and facilitate skeletal muscle adaptations to training [9,10,11]. Specifically, given its role in skeletal muscle protein turnover, dietary protein may enhance the adaptive processes to both strength and endurance training [12]. Skeletal muscle adaptations depend on the balance between muscle protein breakdown (MPB) and protein synthesis (MPS) with skeletal muscle accretion requiring MPS to be greater than MPB. Following exercise, MPS and MPB are elevated in the fasted state but, without adequate feeding, muscle protein balance remains negative [13,14]. Research to date has demonstrated that 40 g of protein is sufficient to maximise MPS post-exercise [15]. Furthermore, MPS is an energy-demanding metabolic process and, therefore, an adequate supply of energy intake (EI) needs to be consumed per day [16]. As such, an energy surplus of ~358–478 kcal·day^−1^ is recommended when undertaking training to support skeletal muscle hypertrophy and minimize fat mass gains [17].

Recent evidence highlighted that male recruits during British Army BT may have insufficient energy intake (EI) at 69% of the Military Dietary Reference Values (MDRVs), with relative protein intakes at 1.5 ± 0.3 g·kg^−1^·d^−1^ [18] which is less than the current sport nutrition recommendations (1.7–2.2 g·kg^−1^·d^−1^) [10]. It is not known, however, if current recommendations reflect the minimum amount of protein needed to maintain nitrogen balance and meet biological needs, or if they are optimal for modulating adaptations to BT. The Scientific Advisory Committee for Nutrition (SACN) states that British Army recruits should consume 12–15% of EI as protein. Based on a typical body mass in this population for men (74.6 kg), this corresponds to a relative intake of 1.6–2.0 g·kg^−1^·d^−1^ [18]. To date, no study has assessed protein requirements in British Army recruits undergoing BT to determine if nutritional interventions are required to optimise dietary patterns, particularly protein intake, that may improve training adaptations. The existing literature has attempted to quantify protein requirements in military populations in the United States (U.S.). These have only assessed requirements over acute periods (days) and have focused on requirements following field operations and in the postabsorptive state only, which may not reflect protein requirements over an entire day [19]. Failure to meet dietary protein requirements may contribute to suboptimal lean body mass accretion and strength adaptations [20], possible immunosuppression [21], and may increase risk of musculoskeletal injury [12,22]. Furthermore, no study to date has assessed changes in dietary intake and nitrogen balance during British Army BT to identify if nutritional interventions need to be implemented and when.

Protein requirements are traditionally estimated using the urinary nitrogen balance method [20,23,24,25,26,27,28] and provides an estimation of total body protein balance [20,25]. In the athletic literature, protein requirements of endurance and strength athletes have been estimated to be ~1.6–1.8 g·kg^−1^·d^−1^ [20,24,29]. Additionally, protein requirements may be as high as 2.4 g·kg^−1^·d^−1^ in individuals who are energy restricted [16], which is typically observed in individuals during prolonged military training [30]. It is suggested that the protein requirements during military training, which combines strength and endurance training concurrently, are >1.5 g·kg^−1^ d^−1^ [31], but no study to date has established this using empirical evidence of nitrogen balance with habitual protein intakes in British Army recruits.

The aims of this study were: (i) compare habitual dietary intake to recommendations and to determine the change in energy and macronutrient intake in male British Army infantry recruits undergoing BT; (ii) measure urinary nitrogen balance to estimate habitual protein requirements during BT. It was hypothesised that participants would not meet nutrient recommendations and that energy and macronutrient intake would not significantly change during BT as military populations undergoing training typically do not meet dietary recommendations [18,30]. It was also hypothesised that participants would not be in nitrogen balance during BT based on previously recorded daily protein intakes in this population [18].

## 2. Materials and Methods

### 2.1. Ethical Approval and Study Design

This study was approved by the U.K. Ministry of Defence Research Ethics Committee and was conducted in accordance with the declaration of Helsinki. This prospective cohort study with repeated measures aimed to collect data at the beginning, mid and end of BT.

### 2.2. Participants and Training Schedule

Potential participants were invited to take part in the study by day 7 of BT following initial inductions in week 1. Data were collected in weeks 2, 6 and 11 as these all included typical BT activities (Table 1) and were deemed to represent the typical training weeks of BT. Additionally, due to the practical constraints of data collection during BT these weeks were chosen to assess and represent dietary intake and nitrogen balance during the start, mid and end of BT. Nineteen men at the infantry Training Centre, Catterick (ITC(C) volunteered and undertook the standard Combined Infantry course. The participants were all part of the same training platoon completing the same training course. The participants also had to all pass the same entry standards suggesting physical demographics and training status amongst individuals was homogenous. In total, 10 participants (mean ± SD: age 20.3 ± 3.1 years., height: 175.2 ± 5.1 cm, body mass 78.2 ± 9.5 kg) completed the study and followed a standardised 14-week Combined Infantry course with set training hours of 0600–2200h. In addition to the activities within the course participants were also required to march to each activity and location around the training centre. In week 2, the participants also completed the start of training performance tests which consisted of a seated medicine ball throw, mid-thigh pull and 2-km run.

### 2.3. Height and Body Mass Measurements

Participant’s height (cm) and body mass (kg) were collected in each week using a seca 213 mobile stadiometer and pre-calibrated seca flat scales (Hamburg, Germany) with participants wearing Army uniform except for boots (with uniform taken into consideration). All uniforms were estimated to weigh 0.5 kg which was subtracted from the original participant’s body mass.

### 2.4. Diet Logs

Dietary assessment was conducted in weeks 2, 6 and 11 of BT. Data were then analysed for 4 consecutive days (Monday to Thursday) across each week. Dietary intake was recorded using researcher-led food weighing at breakfast, lunch and dinner in the training centre dining facility. On arrival, participants chose their food and each portion was weighed using pre-calibrated food scales (Salter, 1066 BKDR15, Kent, UK). After each meal, participants were instructed to leave food discards so that these could be weighed and subtracted from the original weight to give the actual food portion consumed for each meal [32]. To capture dietary intake between meals and while off-camp, participants completed food diaries estimating portion size using subjective measures (1 cup, 2 handfuls, 1 palm size, etc.) [33] and kept any snack or ration discards in discard bags to check against food diaries. Participants were briefed on how to accurately complete a food diary and these were then checked by a member of the research team every day during collection periods to clarify any unclear information.

### 2.5. Nutritics Analysis

Weighed food records and food diary records were entered into nutritional analysis software (Nutritics, Dublin, Ireland) to generate mean daily energy and macronutrient intakes using the UK SACN database. Recipes that did not exist in the database (i.e., ration pack foods) were manually entered using the recipe or nutritional content information provided by the caterer. All data were inputted by the same researcher to reduce data processing variability [34].

### 2.6. Nitrogen Balance

Participants were issued 3-litre urine containers whereby 24-h urine volumes were determined towards the end of weeks 2, 6 and 11 of BT. Participants were instructed to collect all urine during the 24-h period except the first void, in line with previously reported research [24,29,35,36]. Following collection, and once total urine volume had been determined, 2 millilitre aliquots were collected in duplicate to assess intra-sample variability. Samples were then frozen and stored at −80 °C until analysis. Urinary urea nitrogen excretion was assessed to determine nitrogen balance using the below equation [37,38] with protein intake (g) assumed to be 16% nitrogen and miscellaneous nitrogen excretion assumed to be 4 g [38]:

Nitrogen balance (g) = total nitrogen intake (g)—urinary urea nitrogen (g) + 4 (1)

All urinary urea analysis was conducted by the Core Biochemical Assay Laboratory, Cambridge, U.K. Urinary nitrogen was determined enzymatically with a clinical chemistry system (Siemens Healthcare Diagnostics Ltd., Newark, DE, USA). The intra-sample coefficient of variation was 0.8% which indicates a low intra-sample variability and, therefore, high intra-sample reliability.

### 2.7. Statistical Power and Data Analysis

A standard G*Power (Dusseldorf, V 3.1) function was used to estimate sample size for a repeated-measures (within-subjects) analysis of variance (RM ANOVA) to statistically determine differences in absolute protein intake and nitrogen balance at weeks 2, 6 and 11 of BT. With an estimated 10% probability (power = 0.90) of a type-II error, an alpha level of 0.05 and an effect size (η_p_^2^) of 0.32 [18] and 0.75 [38] for absolute protein intake and differences in nitrogen balance, respectively, a total sample size of seven participants was sufficient to detect significant differences from zero.

Participants who did not complete diet logs and 24-h urine collections in each week were not included in the data analyses. Overall, the data sets of 10 participants were included in the data analyses with the remaining nine participants being either back trooped or discharged from BT, and therefore excluded from the analyses. Absolute energy and macronutrient intakes were compared to MDRVs [39] and macronutrient intakes relative to body mass were compared to sport nutrition guidelines [10]. The relative change in nitrogen balance between weeks was calculated to show the change in nitrogen balance through training.

Statistical analyses were carried out using the Statistical Package for Social Sciences, v26 with significance set at *p* ≤ 0.05. The absolute and relative nutrient intakes, and absolute nitrogen intake, nitrogen excretion and nitrogen balance are presented as means and standard deviations in the main text and tables. Data in figures are presented as mean and individual data to demonstrate variance. Data were compared between time points for each participant using a series of RM ANOVA with Bonferroni post hoc comparisons with effect sizes (*d*) [40] calculated and presented. Additionally, nitrogen intake and excretion were compared in each week using a paired samples *t*-test to further clarify nitrogen balance. In cases where the assumption of sphericity was violated, the Greenhouse–Geisser correction factor was applied. Where data showed a significant deviation from a normal distribution, a non-parametric equivalent (Friedman’s ANOVA) was used. Post-hoc analysis was used with Wilcoxon signed-rank tests conducted with a Bonferroni correction applied.

## 3. Results

### 3.1. Energy and Macronutrient Intake

A total of ten male participants (mean ± SD: age 20.3 ± 3.1 years, height: 175.2 ± 5.1 cm, body mass 78.2 ± 9.5 kg) which was sufficient for statistical power completed the study. Participants did not meet MDRVs for daily absolute energy (kcal) and CHO (g) intake in all weeks, nor did they meet the recommended daily protein (g) intake in week 11 of BT (Table 2). The relative (%) intake of CHO did not meet MDRVs in week two and six of BT. When data were expressed relative to body mass and compared to sport nutrition guidelines, the daily CHO and protein intake did not meet recommendations in all weeks.

A significant main effect for absolute (g) protein intake (F(_2,18_) = 7.312, *p* = 0.01, η_p_^2^ = 0.448) between weeks was observed. Further post hoc analysis revealed that absolute protein intake was significantly greater in week 2 compared to week 11 (*p* = 0.02, ES = 1.26). There were no significant main effects observed for mean absolute daily energy (F(_2,18_) = 0.862, *p* = 0.44, η_p_^2^ = 0.087), fat (F(_2,18_) = 0.25, *p* = 0.79, η_p_^2^ = 0.027) or CHO intake (X^2^ (2) = 5.600, *p* = 0.06) between weeks.

A significant main effect was observed for relative (%) fat (F(_2,18_) = 4.364, *p* = 0.03, η_p_^2^ = 0.327), CHO (X^2^(2) = 11.128, *p* < 0.01) and protein intake (X^2^ (2) = 14.600, *p* < 0.01) between weeks. Further post hoc analysis revealed that relative fat intake was not significant between weeks (*p* value range: 0.11–1.00). CHO intake was significantly greater in week 2 compared to week 11 (Z = −2.497, *p* = 0.01, ES = −1.49). Relative protein intake was greater in week 2 compared to week 11 (Z = −2.803, *p* = 0.01, ES = 2.24) and in week 6 compared to week 11 (Z = −2.599, *p* = 0.01, ES = 1.28).

There was no significant main effect for daily CHO (F(_2,18_) = 2.870, *p* = 0.08, η_p_^2^ = 0.242) or fat intake (F(_2,18_) = 0.281, *p* = 0.76, η_p_^2^ = 0.030) when normalised for body mass (g·kg^−1^). A significant main effect was observed for normalised protein intake between weeks (F(_2,18_) = 6.034, *p* = 0.01, η_p_^2^ = 0.401). Further post hoc analysis indicated no significant differences between weeks (*p* value range: 0.06–1.00). However, between weeks 2 and 11 differences in protein intake came close to significance (*p* = 0.06, ES = 1.18).

### 3.2. Body Mass and Urine Analysis

**Figure 1:**
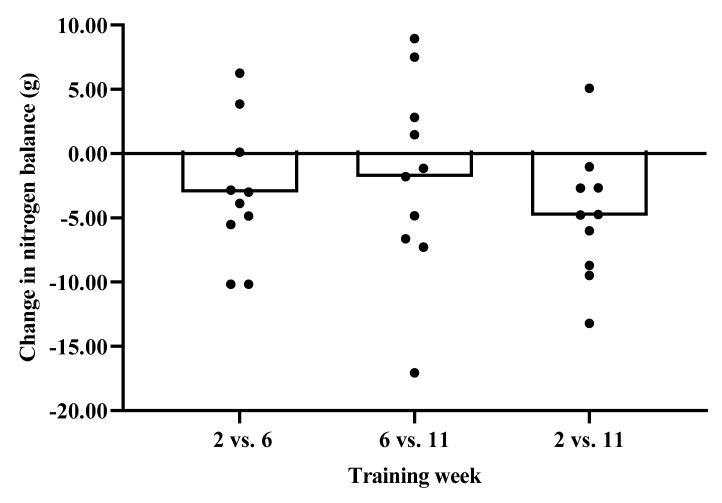
Data presented as mean change (g) in nitrogen balance between weeks. Individual data are also represented.

**Figure 2:**
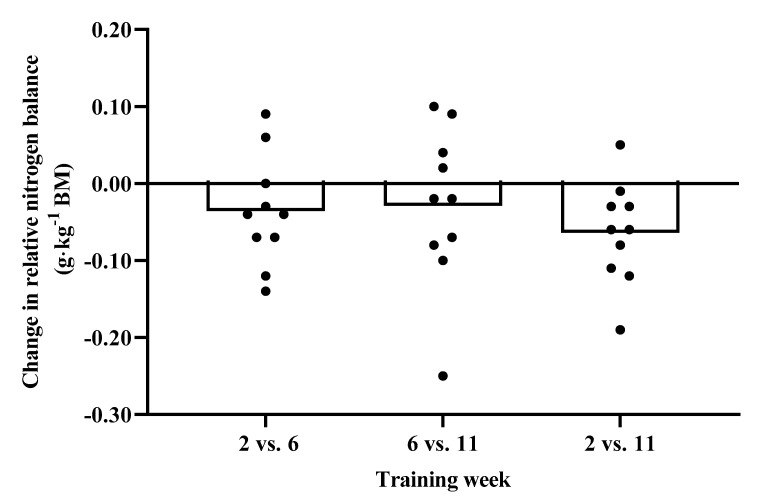
Data presented as mean change (g·kg^−1^ BM) in nitrogen balance between weeks. Individual data are also represented.

There was no significant main effect for body mass (F(_2,18_) = 1.276, *p* = 0.30, η_p_^2^ = 0.124), nitrogen balance (F(_2,18_)= 3.152, *p* = 0.07, η_p_^2^ = 0.259) or nitrogen balance relative to body mass (F(_2, 18_) = 3.249, *p* = 0.06 η_p_^2^ = 0.265) between weeks (Table 3). A significant main effect was observed for nitrogen intake (F(_2,18_)= 7.312, *p* = 0.01, η_p_^2^ = 0.448), whereby post hoc analysis indicated that nitrogen intake was greater in week 2 compared to week 11 (*p* = 0.02, ES = 1.00). A significant main effect for nitrogen excretion (F(_2,18_) = 3.455, *p* = 0.05, η_p_^2^ = 0.277) was also observed. Post hoc analysis revealed that nitrogen excretion was greater in week 6 compared to week 2 (*p* = 0.03, ES = 0.72). Nitrogen intake was significantly greater than nitrogen excretion in week 2 (t(_9_) = 3.546, *p* = 0.01) but not in weeks 6 (t(_9_) = 1.111, *p* = 0.30)) or 11 (t(_9_) = −0.115, *p* = 0.91). The changes in urinary nitrogen balance between weeks is shown in Figure 1 and Figure 2, including reference to individual data.

## 4. Discussion

The key findings of this study support our primary hypothesis, in that participants did not meet MDRVs for daily energy intake and did not meet sport nutrition guidelines for daily CHO and protein intake during BT. Fat intake was adequate when compared to the MDRVs with no statistical difference reported between weeks. Our secondary hypothesis was rejected as it was found that participants maintained nitrogen balance during weeks 2, 6 and week 11 of BT. This was due to no statistically significant difference in nitrogen balance between weeks. The lack of statistical significance may be due to the high variability observed in the data despite adequate power. It was found that nitrogen intake was only significantly greater than nitrogen excretion in week 2 which suggests nitrogen balance declined later on in BT. However, it is acknowledged that at no timepoint was nitrogen excretion significantly greater than nitrogen intake, indicating nitrogen balance. Nonetheless, the gradual decline in nitrogen balance may still be physiologically relevant and suggests participants may require a greater provision of protein (>1.5 g·kg^−1^·day^−1^) to meet training demands and optimise training adaptations which is in-line with the current literature [10].

This study has shown that infantry recruits consume significantly less CHO in week 2 compared to week 11 of BT. These results are similar to those observed in previous work, whereby recruits did not meet the MDRVs for energy and CHO intake during BT [18]. The intake of dietary fat observed in this study suggests that the inadequate intake of total energy is predominantly due to suboptimal daily intakes of CHO and protein particularly when intakes of these macronutrients were below recommendations [10]. The CHO intake reported in this study may have physiological implications when viewed in the wider context of other literature. It has been shown that the initial weeks (weeks 1–2) of training expose men to higher training impulses compared to later training weeks in a similar cohort [1]. The significantly lower CHO intake in week two may compromise muscle and liver glycogen storage [11], immune responses to training [41], mood and exercise performance [42] and increased risk of musculoskeletal injury [43]. Therefore, in-line with other research [30], it is proposed that recruits aim for 5–8 g·kg^−1^·day^−1^ of CHO [10] to mitigate against the negative outcomes of inadequate CHO intake. High rates of musculoskeletal injury are reported during the initial weeks of BT [4]. The cause of musculoskeletal injury is multifactorial, yet a lower CHO intake during the initial weeks of BT may further exacerbate the risk of musculoskeletal injury. A recent study conducted by McGinnis et al. [43] found U.S. Army recruits who increased CHO intake were five times less likely to suffer a musculoskeletal injury and four times less likely to miss training when compared to a control group. Moreover, inadequate CHO intake is also a risk factor for stress fracture in male infantry recruits [44]. Mechanistically, an adequate CHO provision has shown to attenuate bone resorption responses, independent of energy availability, in the two hours post-exercise [45,46], and therefore may reduce stress fracture risk [44].

The potential implications of an inadequate CHO intake on training adaptations are likely to be further exacerbated with an inadequate protein intake in relation to training demands [12]. Participants in the current study maintained urinary nitrogen balance but it was observed that nitrogen balance declined through training and nitrogen intake was only significantly greater than nitrogen excretion in week 2. This observation and that participants did not meet sport nutrition guidelines for daily protein intake [10] suggest that British Army infantry recruits require higher protein intakes than that reported in this study. Protein is needed for the remodelling and regeneration process of skeletal muscle after exercise [22,47] which could reduce the risk of musculoskeletal injury during BT [48] and enhance exercise performance [49]. Elsewhere, it has been shown that U.S. Army recruits who consumed additional protein (2.8 g·kg^−1^·day^−1^) had greater improvements in performance and body composition compared to those who consumed additional CHO [50].

To our knowledge, this is the first study to show that participants undertaking prolonged military training require habitual protein intakes of at least 1.2–1.5 g·kg^−1^·d^−1^ to maintain nitrogen balance. It is unlikely, however, that this amount of protein each day will optimise training adaptations. It was observed that nitrogen intake was only significantly greater than nitrogen excretion in week 2 which suggests nitrogen balance declined later on in BT. Furthermore, although nitrogen excretion was not significantly greater than nitrogen intake at any timepoint, indicating nitrogen balance, a decline in nitrogen balance during BT was observed. The overall decline in nitrogen balance during BT suggests recruits require greater protein provision to meet the demands of training. Failure to maintain nitrogen balance may result in the loss of lean mass as well as suboptimal adaptations to training [13,20]. Data in this study suggest that participants could be at a greater risk of injury and suboptimal physical performance during training due to the decline in urinary nitrogen balance which reflects a decline in whole-body protein balance [26]. To date, the evidence for the benefits of an additional protein intake on training adaptations during military training is scant and has mainly been conducted in U.S. military populations [43,48,50]. Therefore, future research investigating additional protein intake and its influence on training adaptations should be considered as a potential strategy to promote training outcomes.

It is acknowledged that protein requirements can be estimated using other methodologies to that used in this study, including the amino acid tracer method [27,51]. The amino acid tracer method can be used to estimate changes in MPS and MPB over acute periods which, compared to the nitrogen balance method, may be more useful at estimating protein requirements for optimising MPS and therefore training adaptations. It is noted, however, that in the athletic literature, studies which have used the nitrogen balance method to estimate protein requirements [24] have estimated protein requirements to be similar to other studies that have used the amino acid tracer method [51]. Despite being more sensitive at measuring changes in MPS [52], the amino acid tracer method still has limitations. These include the assumption that the metabolism of one amino acid, typically phenylalanine, represents all other amino acids [26] with discrepancies in physiological responses being observed when multiple amino acids are traced [53]. Furthermore, protein requirements estimated using the amino acid tracer method may be influenced by an individual’s habitual protein intake and subsequently result in an overestimation of the amount of protein needed per day to meet training demands [54]. Considering the limitations of measuring protein metabolism, it is acknowledged that assessing protein requirements during military training is challenging and each method has its limitations. Therefore, more research is needed to better determine the effects of prolonged military training on protein metabolism and further studies using both the nitrogen balance and amino acid tracer methodology will likely improve our understanding of how varying protein intakes influence changes in whole-body protein balance and training adaptations.

This study is not without its limitations. Firstly, only male participants were included, therefore the protein requirements in women during British Army infantry BT remain unknown and should be investigated as women are at a greater risk of musculoskeletal injury compared to men during BT [6]. Secondly, this study used the nitrogen balance method which has been suggested to underestimate protein requirements [55] due to an underestimation of nitrogen excretion [51]. This method, however, has been shown to estimate similar protein requirements in athletes to studies which have used other methods, such as the amino acid tracer methodology [51]. Notwithstanding, these limitations this study provides novel data into the protein requirements during prolonged military training.

## 5. Conclusions

This is the first study to investigate the changes in energy and macronutrient intake, and urinary nitrogen balance during British Army infantry BT. Energy, CHO and protein intakes of infantry recruits do not meet recommended values with absolute protein intakes significantly lower in week 11 compared to week two of BT. In some individuals, training may have a deleterious effect on urinary nitrogen balance as indicated by an overall decline in nitrogen balance during training. This may have implications on training adaptations during BT due to suboptimal protein intakes. Recruits may require 1.5 g·kg^−1^·d^−1^ of protein to maintain nitrogen balance in the early stages of BT but higher intakes may be needed later in training. It is also not known if protein intakes above 1.5 g·kg^−1^·d^−1^ provide greater physiological adaptations to training in this population. Therefore, future research investigating the effects of an increased protein intake (>1.5 g·kg^−1^·d^−1^) on urinary nitrogen balance to optimise training adaptations in military populations is warranted.

## Figures and Tables

**Table 1:** The training activities completed by the participants in weeks 2, 6 and 11. Minutes·week^−1^ = minutes per week; hours·week^−1^ = hours per week.

Activity	Week 2	Week 6	Week 11
Rifle practice (minutes·week^−1^)	560	780	1560
Agility training (minutes·week^−1^)	100		
Swimming (minutes·week^−1^)	30	80	
Field exercise (hours·week^−1^)	24		
Foot drill (minutes·week^−1^)	200	100	220
Obstacle course (minutes·week^−1^)		100	
6-km 15 kg loaded march (minutes·week^−1^)		100	
Resistance training (minutes·week^−1^)			100
2-km 15 kg loaded march (minutes·week^−1^)			15
4-km 25 kg loaded march (minutes·week^−1^)			50
Battle physical training (minutes·week^−1^)			100

**Table 2:** The energy and macronutrient intake of participants at weeks 2, 6 and 11 of BT compared to MDRVs (absolute and relative (%)) and sport nutrition guidelines (relative (g·kg^−1^·day^−1^)). All data are presented as mean ± SD.

Nutrient	Recommendation	Week 2	Week 6	Week 11
Energy (kcal)	4100–4600	2677 ± 490	2979 ± 649	2838 ± 403
CHO (g)	513–575	280 ± 92	353 ± 106	357 ± 57
Protein (g)	103–115	115 ± 18 ^a^	116 ± 19	91 ± 20 ^a^
Fat (g)	114–128	121 ± 23	122 ± 22	115 ± 16
CHO (%)	50–65	41 ± 8 ^a^	47 ± 5	50 ± 3 ^a^
Protein (%)	10–15	17 ± 3 ^a^	15 ± 3 ^b^	12 ± 1 ^a,b^
Fat (%)	25–35	41 ± 5	37 ± 3	36 ± 3
CHO (g·kg^−1^·d^−1^)	5.0–8.0	3.7 ± 1.4	4.7 ± 1.6	4.7 ± 0.6
Protein (g·kg^−1^·d^−1^)	1.7–2.2	1.5 ± 0.3	1.5 ± 0.3	1.2 ± 0.2
Fat (g·kg^−1^·d^−1^)	0.5–1.5	1.6 ± 0.4	1.6 ± 0.4	1.5 ± 0.2

g = grams, % = percentage of energy intake, g·kg^−1^·d^−1^ = grams per kilogram of body mass per day. ^a^ denotes a significant difference between week 2 and 11 and ^b^ denotes a significant difference between week 6 and 11 (*p* ≤ 0.05).

**Table 3:** The body mass, nitrogen excretion, nitrogen intake and nitrogen balance of participants in week 2, 6 and 11 of BT. All data is presented as mean ± SD.

Measure	Week 2	Week 6	Week 11
Body mass (kg)	77.7 ± 9.5	76.5 ± 8.8	77.2 ± 8.2
N excretion (g)	13.8 ± 4.2 ^a,^*	16.9 ± 4.4 ^a^	14.8 ± 4.0
N intake (g)	18.4 ± 2.9 ^b,^*	18.5 ± 3.0	14.6 ± 3.2 ^b^
N balance (g)	4.6 ± 4.1	1.6 ± 4.5	−0.2 ± 5.5
N balance (g·kg^−1^)	0.06 ± 0.06	0.03 ± 0.06	−0.01 ± 0.08

N = nitrogen, g = grams, kg = kilograms. ^a^ denotes a significant difference between week 2 and 6, ^b^ denotes a significant difference between week 2 and 11. * denotes significant difference between nitrogen intake and excretion in that week (*p* ≤ 0.05). Nitrogen balance= total nitrogen intake (g)—urinary urea nitrogen (g) + miscellaneous nitrogen losses (assumed to be 4 g) as shown in Equation (1).
